# Association of elevated plasma CCL5 levels with high risk for tic disorders in children

**DOI:** 10.3389/fped.2023.1126839

**Published:** 2023-04-05

**Authors:** Hai-zhen You, Jie Zhang, Yaning Du, Ping-bo Yu, Lei Li, Jing Xie, Yunhui Mi, Zhaoyuan Hou, Xiao-Dong Yang, Ke-Xing Sun

**Affiliations:** ^1^ Department of Traditional Chinese Medicine, Shanghai Children’s Medical Center, Shanghai Jiao Tong University School of Medicine, Shanghai, China; ^2^ Department of Biochemistry and Molecular Cell Biology, Shanghai Jiao Tong University School of Medicine, Shanghai, China; ^3^ Shanghai Institute of Immunology, Department of Immunology and Microbiology, Shanghai Jiao Tong University School of Medicine, Shanghai, China; ^4^ Clinical Research Center, Shanghai Children’s Medical Center, National Children’s Medical Center, Shanghai Jiao Tong University School of Medicine, Shanghai, China; ^5^ The Research Center for Traditional Chinese Medicine, Shanghai Institute of Infectious Diseases and Biosecurity, Shanghai University of Traditional Chinese Medicine, Shanghai, China; ^6^ Center for Traditional Chinese Medicine and Immunology Research, School of Basic Medical Sciences, Shanghai University of Traditional Chinese Medicine, Shanghai, China

**Keywords:** tic disorders, cytokine, biomarker, risk factor, inflammation

## Abstract

Abnormal levels of some peripheral cytokines have been reported in children patients with tic disorders (TDs), but none of these cytokines can be a biomarker for this disease. Our aim was to systemically profile differentially expressed cytokines (DECs) in the blood of TD patients, examine their associations with TD development, and identify from them potential biomarkers for the prediction and management of the risk for TDs. In this study, a cytokine array capable of measuring 105 cytokines was used to screen for DECs in the plasma from 53 comorbidity-free and drug-naïve TD patients and 37 age-matched healthy controls. DECs were verified by ELISA and their associations with TD development were evaluated by binary logistic regression analysis. Elevation of a set of cytokines was observed in TD patients compared with controls, including previously uncharacterized cytokines in tic disorders, CCL5, Serpin E1, Thrombospondin-1, MIF, PDGF-AA, and PDGF-AB/BB. Further analysis of DECs revealed a significant association of elevated CCL5 with TD development (*p* = 0.005) and a significant ROC curve for CCL5 as a risk factor [AUC, 0.801 (95% CI: 0.707–0.895), *p* < 0.0001].

**Conclusion:**

This study identifies associations of a set of circulating cytokines, particularly CCL5 with TD development, and provides evidence that high blood CCL5 has potential to be a risk factor for TD development.

**Clinical Trial Registration:**

identifier ChiCTR-2000029616.

## Introduction

Tic disorders (TDs) are childhood-onset neurodevelopmental conditions characterized by sudden, rapid, recurrent, and nonrhythmic motor movements or vocalizations (1), and based on the type of tics and duration of tic symptoms, they can be classified into 3 major groups: Tourette syndrome (TS), chronic tic disorder (CTD) and provisional tic disorder (PTD). As the most common movement disorders in the pediatric population, TDs affect up to 5% children worldwide (2). Previous literature have supported that this disease can cause physical and mental impairments in multiple domains, such as educational attainment, peer relationships, quality of life, and even premature mortality (2, 3). Tics tend to be refractory to medical treatments and non-medical interventions, and most patients experience relapses that often persist into adulthood. Prediction and management of the risk for TD onset and relapse rely largely on biomarkers which, however, are severely lacking (4).

The etiology of TDs appears to be complex and multifactorial. Growing evidence reveals associations between TDs and various immune disorders, including streptococcal infection-included autoimmunity and many other autoimmune diseases, common allergies, asthma, and maternal immune activation (2, 5, 6). A recent large-scale genome-wide pathway analysis indicates an implication of immune-related pathways in TS (7). These findings link dysregulation of immune responses to TD development. As critical effectors and modulators of immune responses, hundreds of cytokines, consisting of interleukins, chemokines and growth factors, have the potential to be involved in TD development, which is favored by the fact that abnormal levels of a few cytokines in the peripheral blood, like TNF-α, IL-12 and IL-1β have been reported in TD patients (8–10), and the knowledge that TS-associated streptococcal infections are certainly able to induce production of various cytokines. Previous studies, however, were limited to a small number of cytokines, leaving many more cytokines uncharacterized. The goal of this study was to use a cytokine array to simultaneously profile plasma levels of over a hundred cytokines in TD patients and controls, characterize differentially expressed cytokines (DECs), and examine their potential associations with TDs.

## Methods

### Study design and participants

53 patients (median age 8, range 3–16 years) with CTD (11), TS (12) or PTD (13) and 37 age-matched healthy children (median age 9, 3–16 years) who passed outpatient physical examination were recruited for this study. The patients were diagnosed in accordance with the DSM-V criteria and evaluated carefully to exclude those who had any known comorbidities, including mental retardation, autism, attention deficit hyperactivity disorder, and those who received medication within 1 year before admission. The tic severity of each patient was evaluated using the Yale Global Tic Severity Scale (YGTSS), a gold-standard, clinician-administered, semi-structured interview (14). A clinician rates motor and vocal tics in terms of number, frequency, intensity, complexity and interference over the preceding week as well as overall related impairment. Items are rated on a scale from 0 to 5, with higher scores indicative of higher tic severity. YGTSS shows moderate to excellent test–retest reliability, good to excellent internal consistency, inter-rater reliability, convergence validity and moderate to excellent discriminant validity (12, 14, 15). The peripheral blood samples were collected within 4 h of admission, and plasma was immediately prepared, aliquoted, and stored at −80°C for analysis.

The study was approved by the Ethics Committee of Shanghai Children's Medical Center (SCMCIRB-K2019080-3), registered at www.chictr.org.cn (ChiCTR-2000029616), and conducted between July 2020 and November 2021. Informed consent and verbal assent (as appropriate) were provided by parents or legal guardians of all subjects. The study was carried out in accordance with the Helsinki Declaration.

### Detection of cytokines in the plasma

Plasma cytokine profiling was performed with a commercial human cytokine array (R&D Systems, Cat. No.: ARY022B) to measure the relative levels of 105 cytokines according to the manufacturer's instructions. Briefly, equal volume of individual plasmas had been mixed evenly for each group of patients and controls, and the resultant 4 sets of mixed plasma samples were subjected to the array analysis simultaneously. DECs detected in TD patients were individually confirmed by using Quantikine enzyme-linked immunosorbent assay (ELISA) kits from R&D Systems [CCL5(DY478), PDGF-AA(DY221), PDGF-BB(DY220)] as described previously (11).

### Statistical analysis

Data regarding subject characteristics are collected at first visit. Summary statistics for continuous variables are assessed by Kolmogorov–Smirnov test and presented by mean ± standard deviation. Unpaired *t*-test were used for normal distribution data, while Wilcoxon test was used for skewed distribution data. Correlations of DECs levels with tic severity that was scored by following the YGTSS were tested by Pearson's correlation test or Spearman's rank correlation test (depending on the distribution of the variables). Due to the significant differences in gender between healthy control group and TD group, a binary logistic regression analysis was performed to predict TD occurrence from gender, CCL5, PDGF-AA in data collection section and results were expressed by estimating odds ratios (OR) with their 95% confidence intervals. The predictive values of the binary logistic regression analysis were determined using receiver operating characteristic (ROC) curve analysis, and the area under the curve (AUC) was calculated accordingly.

All the data were exported to Excel and SPSS statistics version 26.0. Statistical analyses were performed using the SPSS, and GraphPad Prism 7 was used for mapping. Two-tailed tests were conducted to test statistical significance, and the significance level was set at *p *<* *0.05.

## Results

**Figure 1:**
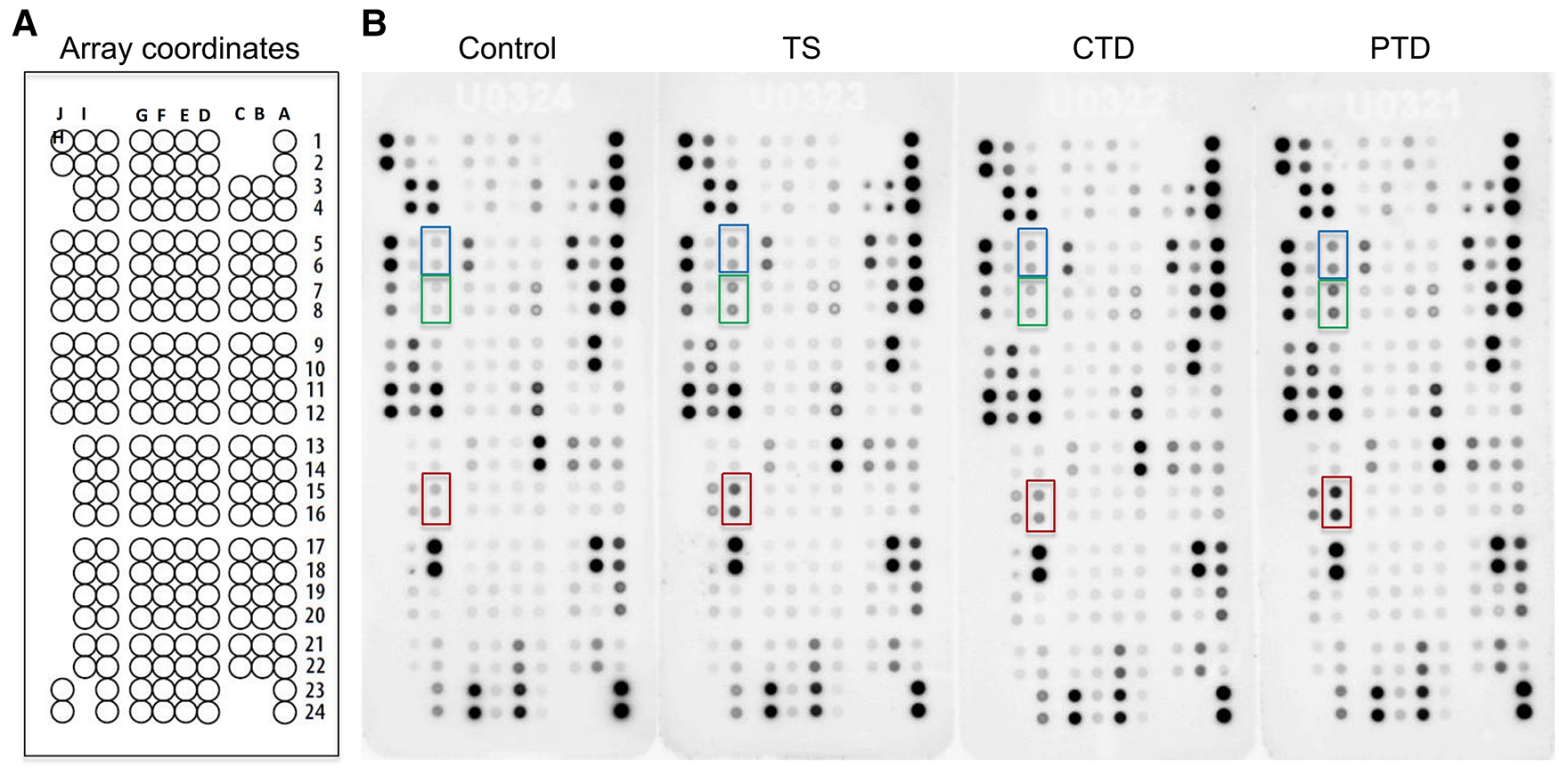
Cytokine array analysis of plasma from patients with tic disorders and healthy controls. (**A**) Schematic diagram of cytokine array coordinates representing 105 different capture antibodies printed in duplicate. (**B**) Array results for control subjects, and TS, CTD, and PTD patients. Dots representing CCL5, PDGF-AA, and PDGF-AB/BB are highlighted in red, blue and green, respectively.

**Figure 2:**
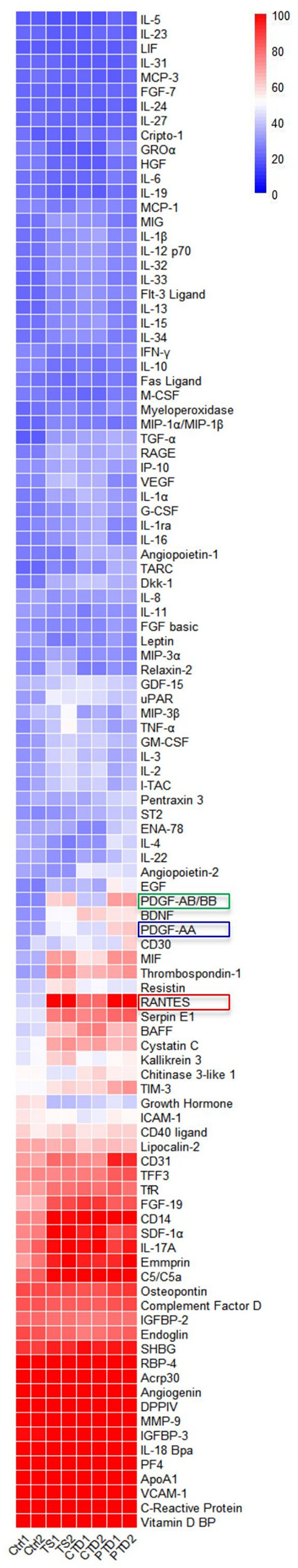
Heat map of original derivation sample, comparisons calculated *via* 1-normal, 2-TS, 3-CTD, 4-PTD. The red, blue, and green boxes are marked with cytokines that differ significantly.

Since comorbidities and prior medications are reported to affect the blood levels of cytokines (10, 16), we excluded comorbid patients and those recently medicated when enrolling tic disorder patients. To systemically compare the relative expression levels of cytokines in TD patients and controls, we employed a cytokine array to profile 105 cytokines (Supplementary Table S1) in the plasma from 3 groups of TD patients and 1 group of control (Table 1). Cytokine signals were developed as spots on film (Figure 1B), and pixel intensity of each spot was quantified for an accurate comparison (Supplementary Table S2). Consistent with previous studies that reported elevation of proinflammatory cytokines, such as TNF-α, IL-12 and IL-1β (8–10), these cytokines were also increased to varying degrees in TD patients in our array assay (Supplementary Table S2). A heatmap analysis of relative levels of all tested cytokines indicated the overall similarities and differences between control subjects and these groups of TD patients (Figure 2). Obviously, the major alterations in the 3 groups of TD patients were elevations of a set of cytokines, including the chemokine RANTES (also known as CCL5), Serpin E1, Thrombospondin-1, MIF, PDGF-AA, and PDGF-AB/BB (a mixture of the B subunit containing PDGF factors detected by antibody to the B subunit that was incapable of distinguishing between AB and BB) (Figure 2), that appeared to be the major DECs.

**Table 1:** General clinical data of healthy control and TD patients.

	Control (*n* = 37)	TD (*n* = 53)	*p*
Age (year)	8.89 ± 3.13 (4–15)	7.91 ± 2.71 (3–16)	0.12
Sex male (%)	17 (45.9%)	41 (77.36%)	0.001*
YGTSS		21.45 ± 7.60	
Motor tics		9.19 ± 2.97	
Vocal tics		4.53 ± 4.79	
Impairment		7.74 ± 4.66	

Values are shown as mean ± standard deviation (SD).

* Statistically significant at *p* < 0.05.

**Figure 3:**
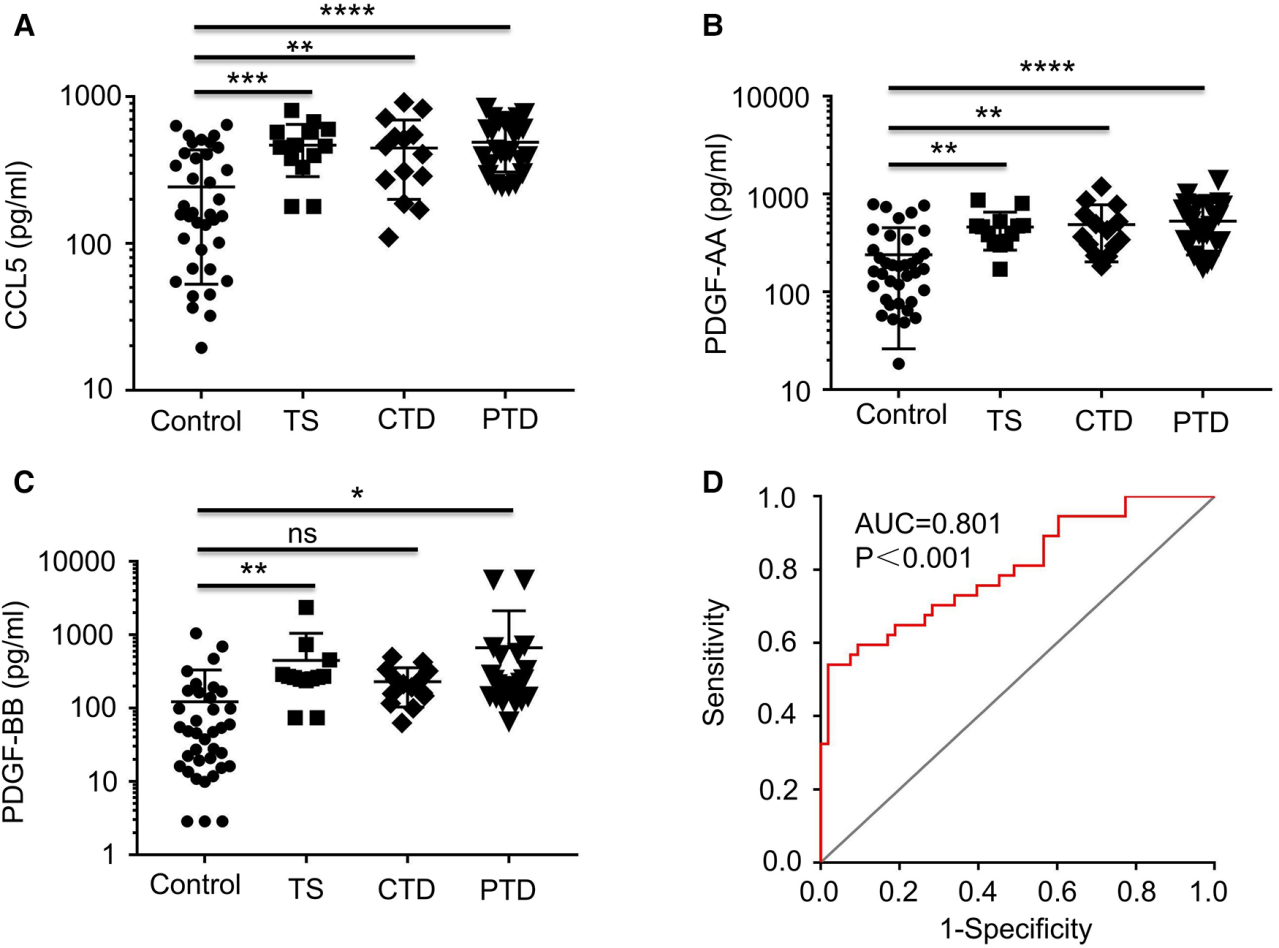
Plots showing the levels of plasma CCL5, PDGF-AA, and PDGF-BB. Plots showing the levels of plasma CCL5 (**A**), PDGF-AA (**B**), and PDGF-BB (**C**) from healthy controls and TD patients (TS, CTD, PTD. (**D**) ROC curve analysis combining CCL5 in the differential diagnosis between TD and normal. ROC, receiver operating characteristic; AUC, area under the curve.

Among the major DECs, CCL5 that ranked in top 3 in all 3 groups of TD patients (Supplementary Table S2) and the two related growth factors, PDGF-AA and PDGF-AB/BB, were selected for further ELISA analysis which confirmed that both CCL5 and PDGF-AA were significantly increased in all 3 groups of TD patients (*p* < 0.02), and PDGF-BB was significantly augmented in TS and PTD patients (*p* < 0.05) but not in CTD patients (Figure 3).

**Figure 4:**
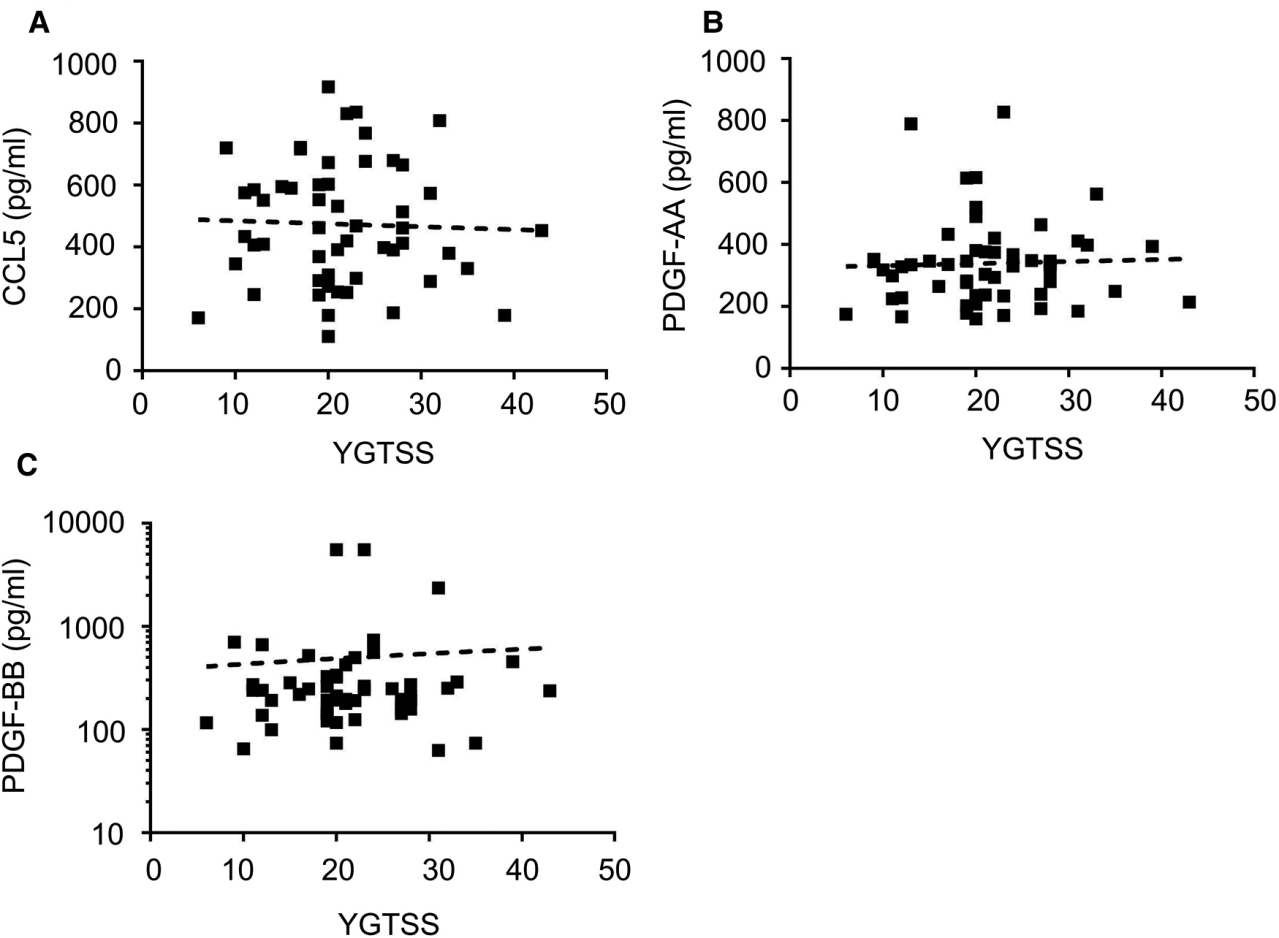
The correlations of the levels of plasma CCL5, PDGF-AA, PDGF-BB with tic severity of all TD patients. The correlations of the levels of plasma CCL5 (**A**), PDGF-AA (**B**), PDGF-BB (**C**) with tic severity of all TD patients assessed with the YGTSS.

Correlation analysis of these 3 cytokines and tic severity of all TD patients assessed with the YGTSS were revealed by Pearson's correlation test, no significant correlation was found for any of these cytokines (Figure 4). The predictive power of these three cytokines and the gender of the subjects that also had a significant difference between patients and controls (Table 1) were evaluated by using binary logistic regression analysis, and the result revealed that both gender (*p* = 0.02) and CCL5 (*p* = 0.005) significantly contributed to TD development (Table 2).

**Table 2:** Analysis of influencing factors in children with TDs.

	OR (95% CI)	*p*
Gender		**0**.02*
female	1.00	
male	3.79 (1.21–11.88)	
CCL5 (pg/ml)	1.005 (1.001–1.008)	**0**.005*
PDGF-AA (pg/ml)	1.004 (0.99–1.01)	0.12

* Statistically significant at *p* < 0.05.

In the case of gender, TD incidence for boys was found to be 3.78 times higher than that for girls in this study (Table 2), which is in line with the reported range of gender preference for TD (17–19). Regarding CCL5, this analysis indicated that an increase of CCL5 concentration by 100 pg/ml caused 50% increase of risk for TD development (OR = 1.005, 95% CI: 1.001–1.008, *p* = 0.005) (Table 2), suggesting a potential for CCL5 to be a TD risk factor. Finally, we combined all TD patients and control subjects for ROC analysis and found that CCL5 had a significant ROC curve [AUC: 0.801 (95% CI: 0.707–0.895), *p* < 0.0001] (Figure 3D). These results suggest CCL5 as a promising predictor for the risk of developing TD.

## Discussion

Aberrant levels of multiple cytokines have been observed in TD patients in previous studies (8–10). However, to our knowledge, none of these cytokines have been characterized as a risk factor of TDs. In this study we used a commercial cytokine array capable of measuring 105 cytokines to unbiasedly profile differentially expressed cytokines in the plasma from comorbidity-free and drug-naïve TD patients and healthy controls and evaluated their associations with TD development. This assay provides the first set of large-scale cytokine profiling data that can be used as a resource for future studies of TDs from the perspective of cytokines. For the first time, we discover CCL5 and PDGF-AA in TDs as two major elevated cytokines in all 3 groups of TD patients and demonstrate their association with TDs. More importantly, we show that while having no correlation with tic severity, peripheral CCL5 has the potential to be a risk factor for evaluating the predisposition to TD development.

Elevation of CCL5 in the blood have been documented in multiple central nervous system (CNS) diseases, such as Parkinson's disease (20), Alzheimer's disease (21), Multiple sclerosis (22, 23), stroke (24), and Traumatic brain injury (25). Interestingly, up-regulated blood CCL5 has also been identified as a risk factor of ischemic stroke that could predict future stroke events (24). These studies, together with ours, reveal a broad association of blood CCL5 with CNS diseases and start to unravel the emerging role of this chemokine in raising the risk for these diseases.

CCL5 is a CC type of chemokine that is widely expressed by many immune cells such as T lymphocytes, macrophages, and platelets. The best known function of CCL5 is to control activation and chemotaxis of many types of immune cells by engaging its cognate receptors expressing on these cells, primarily CCR5, thereby modulating immune responses (26). In the brain, CCL5 and CCR5 are constitutively expressed in astrocytes and microglia and regulate not only chemotaxis of immune cells but also non-immune cell functions (13, 27, 28). It has long been known that chemokines and their cognate receptors are involved in regulation of glial and neuronal cell functions, and that the interactions between glial cells and neurons and through CCR5 and its ligands, e.g., CCL5, are crucial for maintaining neuronal activities, such as neurotransmitter release, ion channel gating and long-term potentiation (27, 29). For instance, after motor neuron injury CCL5 attenuated excessive production of neurotoxic inflammatory mediators in microglia *via* CCR5, and CCR5 deficiency accelerated demise of motor neurons in mice, suggesting that the CCL5/CCR5 pathway plays a neuroprotective role in a manner independent of chemotaxis (30, 31). A later study with a mouse model of Parkinson's disease further demonstrated that CCR5 deficiency resulted in lower numbers of dopamine neurons, reduced levels of striatal dopamine, and decreased locomotor activity (29), indicating that the CCL5/CCR5 pathway plays an important role in maintaining striatal dopamine levels by promoting neuron survival.

The CCL5/CCR5 pathway involved in tic disorders remains unclear so far. Pathologically, tic disorders are considered as a disturbed interplay within and between different brain regions, particularly the basal ganglia-cerebellar-thalamo-cortical network (BGCTC) that functions to inhibit undesired actions. It has been shown that the dysfunction of BGCTC plays a critical role in the pathophysiology of tics (32). Moreover, excessive release of striatal dopamine appears to be the reason for the dysfunction of BGCTC in tic patients (33), and dopamine receptor D2 antagonists that can inhibit dopamine-induced effects represent the most efficacious pharmacotherapy of tics in clinic (34). Thus, it is reasonable to speculate that the CCL5/CCR5 pathway may control tic occurrence by modulating the dopamine neuron-striatal dopamine-BGCTC axis.

Nevertheless, the origin of the elevated blood CCL5 levels is unknown. There are at least two possibilities. One is that blood CCL5 comes from the brain where an inflammatory response is going on and a high level of CCL5 is produced to directly disturb the dopamine neuron-striatal dopamine-BGCTC axis, leading to tic symptoms. Meanwhile, the brain-generated CCL5 can somehow leak into the peripheral blood causing elevation of blood CCL5. The other possibility is that the elevation of blood CCL5 is due to diffusion from other inflamed organs/tissues. It remains to be further studied how the increase of blood CCL5 leads to the risk of functional impairment of brain.

Given that chemokines in the peripheral blood by themselves can hardly cross the blood–brain barrier (BBB) that mainly consists of specialized brain microvascular endothelial cells to control the entry of cells and damaging agents from the blood to brain (35), it is unlikely that low-grade elevation of CCL5 in the blood can directly cause significant detrimental effect to the CNS. Interestingly, CCL5 and its cognate receptors, including CCR5, have been shown to involve in the regulation of BBB permeability and immune cells' entry into the brain. By binding to proteoglycans attached to endothelial cells, CCL5 can be immobilized on endothelial surfaces to enable a high local concentration that facilitates its interaction with the receptors expressed by incoming immune cells in the blood (36). Adhesion of the immune cells to EC-immobilized CCL5 leads to signaling events triggering increase of BBB permeability (35). Increase of BBB permeability presumably exacerbates the access of insults that otherwise cannot penetrate the BBB and enter the brain. In support of the importance of CCL5 and its receptors for BBB regulation, animal studies of epilepsy demonstrated that antagonist-based inhibition of CCR5 on blood cells or blocking CCL5 can reduce BBB permeability and mitigate disease severity (35). A very recent *in vivo* study using highly sensitive radiochemical-based assays showed that circulating CCL5 can be transported across BBB in mice by binding to heparan sulfates at the endothelial surface in a manner independent of CCR2 and CCR5 (37). This finding raises another possibility that circulating CCL5 may exert its regulatory role inside the brain. It is interesting to explore if elevated circulating CCL5 plays a role in reducing BBB permeability or contributing the brain level of CCL5 in the pathogenesis of TDs.

There is evidence that in the brain increased CCL5 can cause pathologic consequences by engaging its cognate receptors CCR5 and CCR1 both of which are expressed on multiple types of cells, such as microglia, astrocytes and neurons (38–41). For example, in a mouse model of intracerebral hemorrhage, CCR5 activation by intracerebroventricularly administrated CCL5 promoted neuronal cell death in the form of inflammatory proptosis, thereby leading to neurological deficits (38). Additionally, CCL5-CCR1-mediated microglial activation in the brain resulted in neurologic deficits and neuroinflammation (42). These animal studies imply that targeting the CCL5-CCR1/5 cascades in the brain could be a promising therapeutic option for neurological diseases associated with CNS.

Based on our findings, we propose that CCL5, and its cognate receptors CCR1/5, could be potential therapeutic targets for TDs. Given the nature of recurring of TDs, pharmaceutical lowering of the level of blood CCL5, attenuation of expression or function of CCR1/5, or inhibition of CCL5-CCR1/5 or CCL5-heparan sulfates interactions, such as by CCL5 antagonist, monoclonal antibodies to CCR1/5, or heparan sulfates competitive inhibitor heparin (37), would hold promise for better risk management of TD relapse.

Due to the limitations to this study that were caused by relatively small numbers and a single cohort of patients, the potential of CCL5 as a risk factor for TD development needs to be validated in multicenter studies of larger cohorts in the future.

## Data Availability

The original contributions presented in the study are included in the article/**Supplementary Material**, further inquiries can be directed to the corresponding authors.

## References

[1] Walkup JT Ferrão Y Leckman JF Stein DJ Singer H. Tic disorders: some key issues for DSM-V. Depress Anxiety. (2010) 27(6):600–10. 10.1002/da.20711 20533370

[2] Fernández de la Cruz L Mataix-Cols D. General health and mortality in tourette syndrome and chronic tic disorder: a mini-review. Neurosci Biobehav Rev. (2020) 119:514–20. 10.1016/j.neubiorev.2020.11.005 33188819

[3] Mi Y Zhao R Sun X Yu P Wang W Li J Sleep disturbances and sleep patterns in children with tic disorder: a case-control study. Front Pediatr. (2022) 10:911343. 10.3389/fped.2022.911343 35979406 PMC9376246

[4] Wang Y Xu X Chen H Zhu M Guo X Gao F. Micro-RNAs from plasma-derived small extracellular vesicles as potential biomarkers for tic disorders diagnosis. Brain Sci. (2022) 12(7):829. 10.3390/brainsci12070829 35884636 PMC9312839

[5] Han VX Patel S Jones HF Dale RC. Maternal immune activation and neuroinflammation in human neurodevelopmental disorders. Nat Rev Neurol. (2021) 17(9):564–79. 10.1038/s41582-021-00530-8 34341569

[6] Spinello C Laviola G Macrì S. Pediatric autoimmune disorders associated with streptococcal infections and tourette's syndrome in preclinical studies. Front Neurosci. (2016) 10:310. 10.3389/fnins.2016.00310 27445678 PMC4928151

[7] Tsetsos F Yu D Sul JH Huang AY Illmann C Osiecki L Synaptic processes and immune-related pathways implicated in tourette syndrome. Transl Psychiatry. (2021) 11(1):56. 10.1038/s41398-020-01082-z 33462189 PMC7814139

[8] Leckman JF Katsovich L Kawikova I Lin H Zhang H Krönig H Increased serum levels of interleukin-12 and tumor necrosis factor-alpha in tourette's syndrome. Biol Psychiatry. (2005) 57(6):667–73. 10.1016/j.biopsych.2004.12.004 15780855

[9] Parker-Athill EC Ehrhart J Tan J Murphy TK. Cytokine correlations in youth with tic disorders. J Child Adolesc Psychopharmacol. (2015) 25(1):86–92. 10.1089/cap.2014.0103 25658821 PMC4340338

[10] Yeon S-M Lee JH Kang D Bae H Lee KY Jin S A cytokine study of pediatric tourette's disorder without obsessive compulsive disorder. Psychiatry Res. (2017) 247:90–6. 10.1016/j.psychres.2016.11.005 27886579

[11] Yang X-D Li W Zhang S Wu D Jiang X Tan R PLK4 deubiquitination by Spata2-CYLD suppresses NEK7-mediated NLRP3 inflammasome activation at the centrosome. EMBO J. (2020) 39(2):e102201. 10.15252/embj.2019102201 31762063 PMC6960439

[12] Storch EA Murphy TK Geffken GR Sajid M Allen P Roberti JW Reliability and validity of the Yale global tic severity scale. Psychol Assess. (2005) 17(4):486–91. 10.1037/1040-3590.17.4.486 16393016

[13] Stuart MJ Baune BT. Chemokines and chemokine receptors in mood disorders, schizophrenia, and cognitive impairment: a systematic review of biomarker studies. Neurosci Biobehav Rev. (2014) 42:93–115. 10.1016/j.neubiorev.2014.02.001 24513303

[14] Leckman JF Riddle MA Hardin MT Ort SI Swartz KL Stevenson J The Yale global tic severity scale: initial testing of a clinician-rated scale of tic severity. J Am Acad Child Adolesc Psychiatry. (1989) 28(4):566–73. 10.1097/00004583-198907000-00015 2768151

[15] McGuire JF Piacentini J Storch EA Murphy TK Ricketts EJ Woods DW A multicenter examination and strategic revisions of the Yale global tic severity scale. Neurology. (2018) 90(19):e1711–9. 10.1212/WNL.0000000000005474 29653992 PMC5952973

[16] Rao NP Venkatasubramanian G Ravi V Kalmady S Cherian A Yc JR. Plasma cytokine abnormalities in drug-naïve, comorbidity-free obsessive-compulsive disorder. Psychiatry Res. (2015) 229(3):949–52. 10.1016/j.psychres.2015.07.009 26187339

[17] Hisle-Gorman E Susi A Stokes T Gorman G Erdie-Lalena C Nylund CM. Prenatal, perinatal, and neonatal risk factors of autism spectrum disorder. Pediatr Res. (2018) 84(2):190–8. 10.1038/pr.2018.23 29538366

[18] Chen S-W Zhong X-S Jiang L-N Zheng X Xiong Y Ma S Maternal autoimmune diseases and the risk of autism spectrum disorders in offspring: a systematic review and meta-analysis. Behav Brain Res. (2016) 296:61–9. 10.1016/j.bbr.2015.08.035 26327239

[19] Liu X Dalsgaard S Munk-Olsen T Li J Wright RJ Momen NC. Parental asthma occurrence, exacerbations and risk of attention-deficit/hyperactivity disorder. Brain Behav Immun. (2019) 82:302–8. 10.1016/j.bbi.2019.08.198 31476415 PMC7408292

[20] Rentzos M Nikolaou C Andreadou E Paraskevas GP Rombos A Zoga M Circulating interleukin-15 and RANTES chemokine in Parkinson's disease. Acta Neurol Scand. (2007) 116(6):374–9. 10.1111/j.1600-0404.2007.00894.x 17986095

[21] Marksteiner J Kemmler G Weiss EM Knaus G Ullrich C Mechtcheriakov S Five out of 16 plasma signaling proteins are enhanced in plasma of patients with mild cognitive impairment and Alzheimer's disease. Neurobiol Aging. (2011) 32(3):539–40. 10.1016/j.neurobiolaging.2009.03.011 19395124 PMC4311051

[22] Bartosik-Psujek H Stelmasiak Z. The levels of chemokines CXCL8, CCL2 and CCL5 in multiple sclerosis patients are linked to the activity of the disease. Eur J Neurol. (2005) 12(1):49–54. 10.1111/j.1468-1331.2004.00951.x 15613147

[23] Sindern E Niederkinkhaus Y Henschel M Ossege LM Patzold T Malin JP. Differential release of beta-chemokines in serum and CSF of patients with relapsing-remitting multiple sclerosis. Acta Neurol Scand. (2001) 104(2):88–91. 10.1034/j.1600-0404.2001.104002088.x 11493224

[24] Canouï-Poitrine F Luc G Mallat Z Machez E Bingham A Ferrieres J Systemic chemokine levels, coronary heart disease, and ischemic stroke events: the PRIME study. Neurology. (2011) 77(12):1165–73. 10.1212/WNL.0b013e31822dc7c8 21849651 PMC3174064

[25] Albert V Subramanian A Agrawal D Bhoi SK Pallavi P Mukhopadhayay AK. RANTES levels in peripheral blood, CSF and contused brain tissue as a marker for outcome in traumatic brain injury (TBI) patients. BMC Res Notes. (2017) 10(1):139. 10.1186/s13104-017-2459-2 28340601 PMC5366123

[26] Marques RE Guabiraba R Russo RC Teixeira MM. Targeting CCL5 in inflammation. Expert Opin Ther Targets. (2013) 17(12):1439–60. 10.1517/14728222.2013.837886 24090198 PMC7103722

[27] Sorce S Myburgh R Krause K-H. The chemokine receptor CCR5 in the central nervous system. Prog Neurobiol. (2011) 93(2):297–311. 10.1016/j.pneurobio.2010.12.003 21163326

[28] Ubogu EE Callahan MK Tucky BH Ransohoff RM. Determinants of CCL5-driven mononuclear cell migration across the blood-brain barrier. Implications for therapeutically modulating neuroinflammation. J Neuroimmunol. (2006) 179(1–2):132–44. 10.1016/j.jneuroim.2006.06.004 16857269

[29] Choi D-Y Lee MK Hong JT. Lack of CCR5 modifies glial phenotypes and population of the nigral dopaminergic neurons, but not MPTP-induced dopaminergic neurodegeneration. Neurobiol Dis. (2013) 49:159–68. 10.1016/j.nbd.2012.08.001 22922220

[30] Gamo K Kiryu-Seo S Konishi H Aoki S Matsushima K Wada K G-protein-coupled receptor screen reveals a role for chemokine receptor CCR5 in suppressing microglial neurotoxicity. J Neurosci. (2008) 28(46):11980–8. 10.1523/JNEUROSCI.2920-08.2008 19005063 PMC6671655

[31] Sorce S Bonnefont J Julien S Marq-Lin N Rodriguez I Dubois-Dauphin M Increased brain damage after ischaemic stroke in mice lacking the chemokine receptor CCR5. Br J Pharmacol. (2010) 160(2):311–21. 10.1111/j.1476-5381.2010.00697.x 20423342 PMC2874853

[32] Ramkiran S Heidemeyer L Gaebler A Jon Shah N Neuner I. Alterations in basal ganglia-cerebello-thalamo-cortical connectivity and whole brain functional network topology in tourette's syndrome. Neuroimage Clin. (2019) 24:101998. 10.1016/j.nicl.2019.101998 31518769 PMC6742843

[33] Caligiore D Mannella F Arbib MA Baldassarre G. Dysfunctions of the basal ganglia-cerebellar-thalamo-cortical system produce motor tics in tourette syndrome. PLoS Comput Biol. (2017) 13(3):e1005395. 10.1371/journal.pcbi.1005395 28358814 PMC5373520

[34] Fernandez TV State MW Pittenger C. Tourette disorder and other tic disorders. Handb Clin Neurol. (2018) 147:343–54. 10.1016/B978-0-444-63233-3.00023-3 29325623

[35] Louboutin J-P Strayer DS. Relationship between the chemokine receptor CCR5 and microglia in neurological disorders: consequences of targeting CCR5 on neuroinflammation, neuronal death and regeneration in a model of epilepsy. CNS Neurol Disord Drug Targets. (2013) 12(6):815–29. 10.2174/18715273113126660173 24047524

[36] Proudfoot AEI Handel TM Johnson Z Lau EK LiWang P Clark-Lewis L Glycosaminoglycan binding and oligomerization are essential for the in vivo activity of certain chemokines. Proc Natl Acad Sci U S A. (2003) 100(4):1885–90. 10.1073/pnas.0334864100 12571364 PMC149928

[37] Quaranta DV Weaver RR Baumann KK Fujimoto T Williams LM Kim HC Transport of the proinflammatory chemokines C-C motif chemokine ligand 2 (MCP-1) and C-C motif chemokine ligand 5 (RANTES) across the intact mouse blood-brain barrier is inhibited by heparin and eprodisate and increased with systemic inflammation. J Pharmacol Exp Ther. (2023) 384(1):205–23. 10.1124/jpet.122.001380 36310035 PMC9827507

[38] Yan J Xu W Lenahan C Huang L Wen J Li G CCR5 activation promotes NLRP1-dependent neuronal pyroptosis via CCR5/PKA/CREB pathway after intracerebral hemorrhage. Stroke. (2021) 52(12):4021–32. 10.1161/STROKEAHA.120.033285 34719258 PMC8607924

[39] Sunnemark D Eltayeb S Wallström E Appelsved L Malmberg A Lassmann H Differential expression of the chemokine receptors CX3CR1 and CCR1 by microglia and macrophages in myelin-oligodendrocyte-glycoprotein-induced experimental autoimmune encephalomyelitis. Brain Pathol. (2003) 13(4):617–29. 10.1111/j.1750-3639.2003.tb00490.x 14655765 PMC8095849

[40] Cowell RM Xu H Galasso JM Silverstein FS. Hypoxic-ischemic injury induces macrophage inflammatory protein-1alpha expression in immature rat brain. Stroke. (2002) 33(3):795–801. 10.1161/hs0302.103740 11872906

[41] Han Y Wang J Zhou Z Ransohoff RM. TGFbeta1 selectively up-regulates CCR1 expression in primary murine astrocytes. Glia. (2000) 30(1):1–10. PMID: . 10696139

[42] Yan J Zuo G Sherchan P Huang L Ocak U Xu W. CCR1 activation promotes neuroinflammation through CCR1/TPR1/ERK1/2 signaling pathway after intracerebral hemorrhage in mice. Neurotherapeutics. (2020) 17(3):1170–83. 10.1007/s13311-019-00821-5 31898284 PMC7609528

